# The Construction of an Aqueous Two-Phase System to Solve Weak-Aggregation of Gigaporous Poly(Styrene-Divinyl Benzene) Microspheres

**DOI:** 10.3390/polym8050142

**Published:** 2016-04-26

**Authors:** Donglai Zhang, Weiqing Zhou, Juan Li, Yace Mi, Zhiguo Su, Guanghui Ma

**Affiliations:** 1 National Key Laboratory of Biochemical Engineering, Institute of Process Engineering, Chinese Academy of Sciences, Beijing 100190, China; dlzhang@ipe.ac.cn (D.Z.); wqzhou@ipe.ac.cn (W.Z.); lijuan@ipe.ac.cn (J.L.); miyacetx@163.com (Y.M.); zgsu@ipe.ac.cn (Z.S.); 2 University of Chinese Academy of Sciences, Beijing 100049, China

**Keywords:** gigaporous microspheres, weak-aggregation, aqueous two-phase system

## Abstract

Gigaporous poly(styrene-divinyl benzene) microspheres made via the surfactant reverse micelles swelling method had a controllable pore size of 100–500 nm. These microspheres had unique advantages in biomacromolecule separation and enzymes immobilization. However, the obtained microspheres adhered to each other in the preparation process. Though the weak aggregation could be re-dispersed easily by mechanical force, it will be difficult to scale up. By analyzing the formation mechanism of the aggregates, a method was presented to rebuild the interface between the internal aqueous channel and the external continuous phase by constructing an aqueous two-phase system (ATPS). Based on the ATPS, the method of emulsification, stirring speed, and surfactant concentration in oil phase were optimized. Under the optimum condition (screen emulsification method, 120 rpm for polymerization and 55% surfactant), the microspheres with a controllable particle size of 10–40 μm and a pore size of about 150 nm were obtained. This new method could significantly decrease the weak-aggregation of microspheres.

## 1. Introduction

Gigaporous microspheres have unique advantages in bioprocess, especially in enzyme immobilization and the purification of biomolecules with large molecular weight and complex structures. Compared to the conventional agarose particles used in bioseparation with pore sizes of 10–30 nm [1,2], gigaporous microspheres have pore sizes up to 100–500 nm [3,4,5,6]. When biomolecules such as PEG-protein, viruses, and VLPs (virus-like particles), whose sizes that are 20 nm or larger are purified [7,8], bigger pores lead to good separation, including higher product bioactivity, a faster separation rate, and higher dynamic loading capacity [9,10,11,12,13]. As the carrier materials used for the immobilization of enzymes, the immobilized lipase in gigaporous microspheres also shows much higher specific activity, thermal stability, and storage stability.

At present, multiple methods are developed to prepare gigaporous polymer materials, such as the high internal phase emulsion method [3,14,15], microfluidity [16], the piling of small particles [17], electrospraying [18], and the double emulsification method [19,20]. However, there are some unfavorable characteristics of the products prepared by these methods, such as low specific surface area and mechanical strength. In order to resolve these disadvantages, a new method named the surfactant reverse micelles swelling method has been put forward [4,5]. This method can prepare polymer microspheres with controllable pore sizes of 100–500 nm and favorable mechanical strength; the yield of this method was up to 95% and is feasible for mass production.

The pore-forming mechanism of this method is as follows. A high concentration of surfactant in oil phase assembles into reverse micelles; and, when the oil phase with micelles mixes with the water phase, the reverse micelles in oil droplets absorb water spontaneously and turn into water channels through the droplet. After polymerization, the water channels become through-holes [21]. By adjusting the surfactant concentration, the diameter of pores can be regulated.

However, a new problem appeared in the preparation process. Some particles aggregated weakly. The aggregation can be easily dispersed at about 0.01 MPa pressure, so we did not think this was a big question at first. However, when the pilot test was done, and the production of gigaporous microspheres reached kilogram grade, great effort, such as ultrasonic dispersion or grind, had to be made to re-disperse the aggregate. Could we avoid the occurrence of these aggregations during the preparation process? Common solutions are adding stabilizer in the continuous phase or increasing the rotation speed of emulsion [22,23]. However, in this system, a high concentration of stabilizer polyvinyl alcohol (PVA) (3%) had been added to the water phase, the viscosity of the water phase was very high. If much more of the stabilizer is added to the water, it is hard to clean the products because of the thick water phase [21]. Moreover, the increase in rotation speed would only have a slight influence to the aggregations of microspheres in this system, and also lead to the changing of the particle size.

Thus, a new method to solve this problem based on the formational mechanism of gigapores was put forward in this study. Generally, the stabilizer adsorbs onto the interface of water and oil [21,23,24]. However, in the reverse micelles swelling process, gigapores made the water in them connected to the external continuous aqueous phase; at this region, the interface disappears and the stabilizer cannot adsorb there. During the polymerization process, the particles will impact each other inevitably. Without protection of the stabilizer, the particles will easily adhere to each other.

If the boundary can be rebuilt, the stabilizer can adsorb onto the interface, and the aggregation may be weakened and even disappear [23]. So rebuilding the boundary is a feasible means of reducing the aggregate.

Because the connected region is in the aqueous phase, we were inspired to establish an aqueous two-phase system (ATPS) to rebuild the phase boundary. The ATPS is established by two incompatible water-soluble substances [25,26,27,28]. When such substances were dissolved in water, a phase separation would occur. Through this method, the occurrence of the aggregation was effectively weakening, and the gigaporous microspheres with good dispersity were fabricated.

## 2. Materials and Methods

### 2.1. Materials

The chemically pure monomer styrene (ST) was purchased from Sinopharm Chemical Reagent Co., Ltd. (Shanghai, China), and the crosslinking agent divinyl benzene (DVB) of commercial grade was obtained from Beijing Chemical Reagents Co., Ltd. (Beijing, China). The Benzoyl peroxide (BPO) with 30% water was used as an initiator and purchased from Sinopharm Chemical Reagent Co., Ltd. (Shanghai, China). Hexadecane (HD) of chromatographic grade was purchased from Haltermann (Houston, TX, USA) and used as a hydrophobic additive. Surfactant sorbitan monooleate (Span 80) of reagent grade was obtained from Farco Chemical supplies (Beijing, China).

Polyvinyl alcohol (PVA), PVA-217, (degree of polymerization 1700, degree of alcoholysis 88.5%), was provided by Kuraray Co., Ltd. (Tokyo, Japan). Polyethylene glycol (PEG) with molecular weights 2000, 6000, 20,000 were purchased from Sinopharm Chemical Reagent Co., Ltd. (Shanghai, China). Dextran T50 (molecular weight about 50,000) were purchased from Seebio Biotech, Inc. (Shanghai, China). NaCl, I_2_, BaCl, and ammonium sulfate [(NH_4_)_2_SO_4_] were purchased from Xilong Chemical Co., Ltd. (Guangdong, China). Ethanol and acetone were used to precipitate and wash the particles, which purchased from Beijing Chemical Works (Beijing, China).

### 2.2. Construction of the ATPS Phase Diagram

The common materials to establish the ATPS are two types: polymer–salt and polymer–polymer. In this research, considered the cost and measurement, the materials of the polymer–polymer were limited to dextran/polyethylene glycol (PEG) and PEG/PVA, and the polymer–salt materials are (NH_4_)_2_SO_4_/PEG or NaCl/PEG.

If turbidness was observed when these substances were dissolved in water, phase separation would spontaneously occur after a certain time, and an ATPS would be constructed. After the ATPS was formed, the phase diagram could then be delineated. When the two phases separated completely, they were respectively extracted, and the concentration of the two phases were measured to determinate the phase separation point (node) and line (tie-line).

The way to determine the concentration of PEG in the ATPS was via UV spectrophotometry because the PEG had an absorbance in 520 nm with the reaction of I_2_ and BaCl, and this absorbance had correlation with the PEG concentration. Therefore, the PEG concentration can be determined by the absorbance–concentration standard curve. When the concentration was determined, the concentration of another component was calculated by the dry weight. After ensuring the node and tie-line, the node was linked with a curve, the binodal of the phase diagram.

### 2.3. Preparation of Polymeric Microspheres

**Figure 1:**
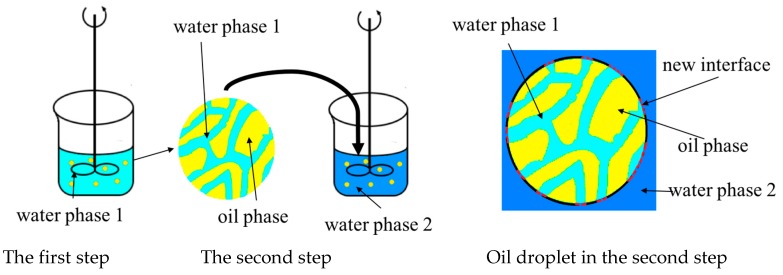
Two-step method to prepare the aqueous two-phase system (ATPS).

A recipe used in previous research [4] of gigaporous particle and materials for an ATPS are shown in Table 1: The mixture of ST (monomer), DVB (crosslinking agent), HD (hydrophobic additive), Span 80 (surfactant), and BPO (initiator) were used as the dispersed phase. Water dissolved with PVA (stabilizer) was used as the continuous phase; on this basis, another component such as PEG or dextran was added into the water phase in order to establish an ATPS. According to the phase diagram, the concentration of each phase in the ATPS could be calculated. In this research, a two-step method was used to construct the ATPS between the inner pore and outside (Figure 1). After preparing each of the aqueous phases respectively, the oil phase was dispersed into one of the aqueous phases to form a first emulsion. A certain amount of time was needed to make the micelle swell. Then, the first emulsion was added into another aqueous phase to obtain the ATPS and then started the process of polymerization with the stirring. The characteristics of the emulsion changed with different emulsification and polymerization conditions, but the use of high concentration PVA made a high viscosity and low interface tension of the emulsion.

The polymerization process occurred at 85 °C for 8 h. The polymeric particles were washed with boiled water, ethanol, and acetone three times in turn and dried at 60 °C for 6 h in a glass dish. The yield of particles was calculated by the weight of dried microspheres and the weight of monomers.

### 2.4. Preparation of Emulsion

In the two-step method, the first step was to make the micelles absorb water and swell, thus determining the size of the oil droplet. In order to get better first emulsion, different methods, including stirring, ultrasonic dispersion, mechanical oscillation membrane emulsification, and screen emulsification methods, were chosen.

For the stirring method, the emulsion was obtained by stirring for 5 to 10 min in 60 to 100 rpm. Additionally, the membrane emulsification method [29,30] was carried out by the use of an SPG membrane, whose pores were from 1 to 50 μm. When the continuous phase and dispersed phase mixed together and were pressed through the membrane by the pressure of the gas, a uniform emulsion could be obtained. Screen emulsification was the same as membrane emulsification, except the SPG membrane was replaced with a large-hole screen.

The second emulsion was prepared by stirring in 120 rpm when the first emulsion was added into another aqueous phase of the ATPS.

### 2.5. SEM Observation

The aggregation, surface feature, and diameter of the microsphere could be observed by the JSM-6700F scanning electron microscope (SEM) (JEOL, Tokyo, Japan). The dry microspheres in the glass dish were used as a sample; these samples were adhered to the double-sided conductive adhesive tape and then placed on the metal stub. In order to observe, the samples were coated with thin gold film below 5 Pa with a JFC-1600 fine coater.

### 2.6. Measurement of Particle and Pore Size Distribution

The average diameter of the microsphere and size distribution was measured by laser diffractometry using Mastersizer 2000E (Malvern Instruments Ltd., Malvern, UK).

The pore size distribution was measured by mercury porosimetry measurements—AutoPore IV 9500 mercury porosimetry (Micromeritics, Norcross, GA, USA).

## 3. Result and Discussion

### 3.1. Establishment of the ATPS

**Figure 2:**
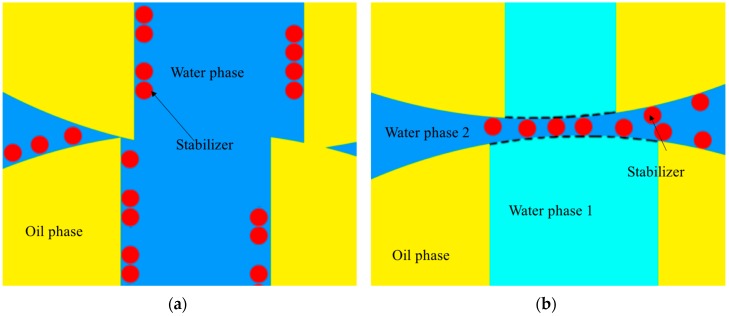
The schematic photographs of boundary loss (**a**) and boundary rebuild (**b**).

When the reverse micelles absorbed the water to form a water channel, the phase boundary, which the stabilizer could not absorb on, was lost, and the aggregation occurred. If the water phase inside and outside the channel formed the ATPS, a new boundary would be rebuilt to allow the stabilizer to adsorb, and the aggregation might decrease and even disappear (Figure 2).

Some factors could influence the properties of the ATPS. The materials were the most significant factor. Common materials to establish the ATPS have two types: polymer–salt and polymer–polymer. Comparing the two types, the polymer–salt type usually had a high salt concentration in one phase. High ionic concentration would make the stabilizer coagulation in our experiment. Thus, the polymer–polymer ATPS became a better choice for preparing the gigaporous particles.

For the polymer–polymer ATPS, PEG/dextran or PEG/PVA was chosen. Because PVA is the original component (stabilizer) of the polymerization system, it is more suitable to select PVA than dextran. Another reason is that PVA is much cheaper than dextran when taking into account the cost of production. The molecular weight of PEG was chosen from three different molecular weights (*M*_w_ = 2000, 6000 and 20,000). Higher molecular weight led to high viscosity of the water phase; meanwhile, it also formed the ATPS with less concentration. In consideration of the PVA concentration to the reaction, the final ATPS component is PEG 20,000 and PVA 217. These two polymers could form the ATPS with an appropriate concentration and a suitable viscosity.

**Figure 3:**
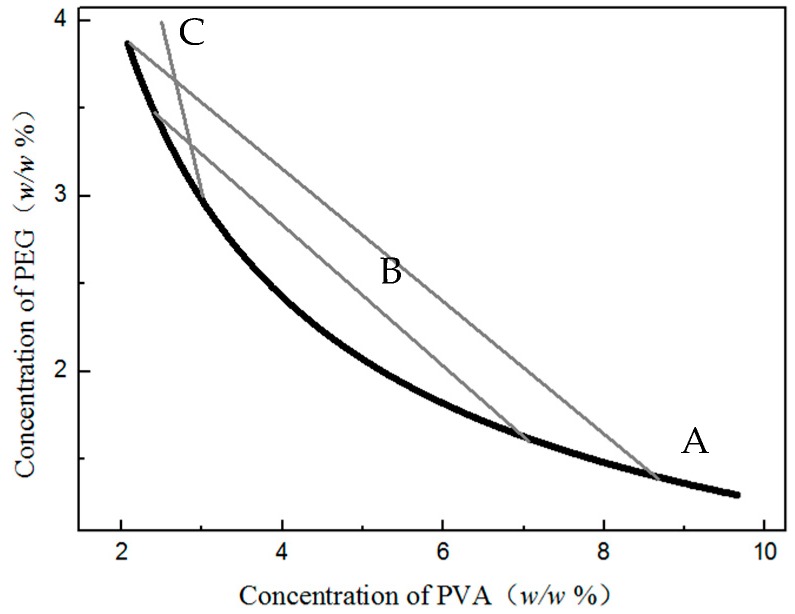
Phase diagram of the polyethylene glycol/polyvinyl alcohol (PEG/PVA) ATPS in 85 °C (A: binodal line; B: tie-lines; C: the concentration ratio chosen in the research).

After choosing the suitable materials, we began to draw the phase diagram. This diagram would provide important guidance in selecting suitable content for the ATPS used in preparing the particle. The temperature for making the ATPS phase diagram is a question that needs to be considered. At room temperature, the swelling step proceed and the boundary lost, and at the polymerization temperature (85 °C) was the time of aggregation. By comparing the phase diagrams made under two different temperatures, we found there was little difference. Therefore, the phase diagram of the PEG/PVA ATPS in 85 °C was chosen for further application (Figure 3). Through this diagram, the concentration and the volume of each phase could be ensured.

### 3.2. Fabrication of Microsphere through the ATPS

In order to rebuild the phase boundary at the droplet, primarily, the interface of the ATPS should close to the initial oil/water interface (Figure 2b), which means that the volume of the aqueous phases inside the oil droplet needs to correspond approximately to the volume of the water channels (the pore volume of the polymerized particles). Furthermore, the volume of the external water phase should be determined by the optimal ratio of the inner phase to the outer phase.

Thus, through calculation, the volume of external water was about 9 times the internal volume. According to the phase diagram, the volume ratio could be determined by the segmentation of tie-line. (Figure 3) In consideration of the stabilization effect and the ease of the after-treatment of the products, the concentration of PVA would be about 3% of the total continuous phase. Therefore, the system concentration of the water phase (*C*_total_) and the concentration in the two water phases (*C*_inner_ and *C*_outer_) can be deduced from the figures in Table 2.

When the concentration of each phase was confirmed, the microspheres could be fabricated. By the two-step method, the phase boundary could be established theoretically. In our experiment, we measured the concentration ratio of the PEG/PVA in the outside water, and the result was nearly 1.5, which was in agreement with the theoretical values of *C*_outer_ and bigger than *C*_total_. This result means that the concentrations of the inner phase and outer phase were not the same (if they were the same, the concentration would be *C*_total_), and confirms the formation of the ATPS.

**Figure 4:**
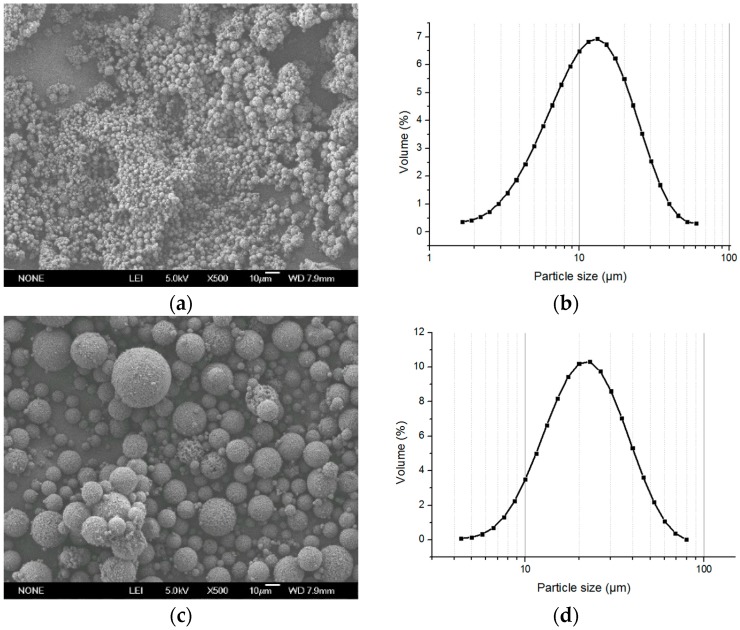
The photographs of the microspheres made at different stirring speeds. Stirring speed 150 rpm, swelling time 10 min (**a**) SEM image (×500) and (**b**) particle size distribution (dispersed). Stirring speed 70 rpm, swelling time 10 min (**c**) SEM image (×500) and (**d**) particle size distribution (dispersed).

**Figure 5:**
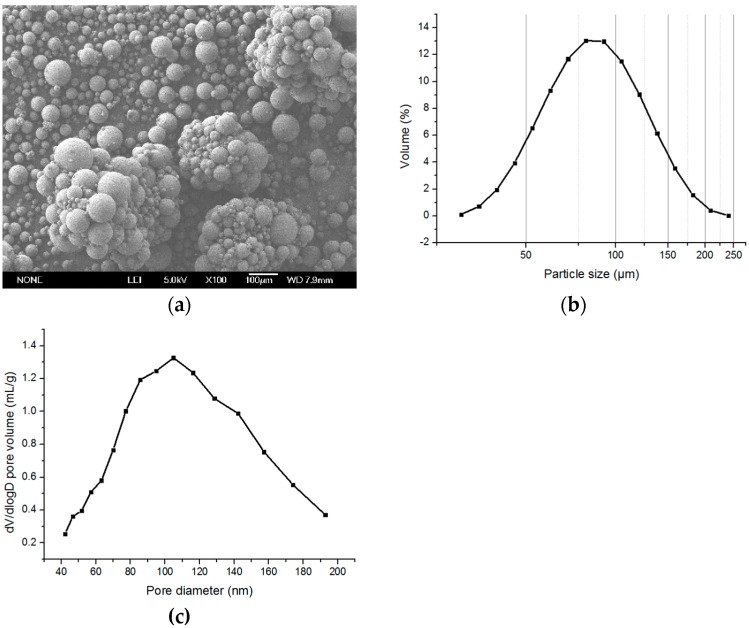
(**a**) The SEM image (×100), (**b**) size distribution (dispersed), and (**c**) pore distribution of control group.

After polymerization, the SEM photo showed that the particle size was one tenth of the microspheres made by the original method (Figure 4a,b), and, for the small microspheres with a diameter of about 10 μm totally aggregated, the aggregation was difficult to disperse so that the size distribution was larger than could be observed. The yield of the reaction was 87% because the small particles were lost in the after-treatment. In addition to the interface effect, another main factor for the aggregation was due to the bigger surface energy of the small particle. Thus, we had to adjust the particle size. Considering that the stirring speed had a significant influence on the particle size, the polymerization was processed with a speed from 150 to 70 rpm. The particle size was from 15 to 25 μm and an aggregated degree was reduced compared with the control group prepared with the same recipe but without the formation of the ATPS (Figure 4c,d and Figure 5). However, we should ascertain whether the existence of the aggregation was due to the small particle or not. Therefore, the next work was aimed at the optimization of the particle size and porous structure of the microspheres in order to find the effect of the ATPS when ruling out the influence of small particles.

### 3.3. Optimization of the Particle Size and Porous Structure of the Microspheres

**Figure 6:**
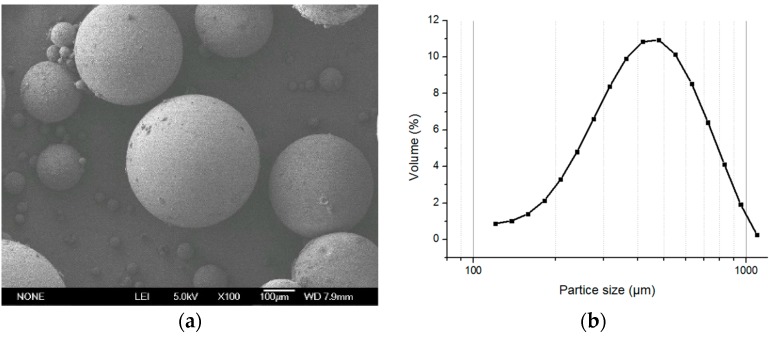
(**a**) The SEM photographs of the microspheres with stirring speed 70 rpm, swelling time 5 min, (×100) and (**b**) size distribution.

As mentioned above, the significant influence of the stirring rate to the particle size was revealed. Moreover, when the time of the swelling process reduced to half (10 to 5 min), the particle size grew from 25 to 350 μm (Figure 6) and the aggregations were prevented. Therefore, both the stirring speed and swelling time had significantly influenced the particle size (Table 3). Comparing the small droplets with the high surface energy intended to aggregate, the big droplet with lower energy was hard to aggregate. However, the pore size of the particle was only around 100 nm (less than one in a thousand of the particle size), and some of the big particles were hollow. Therefore, emulsion with a proper droplet size was needed.

**Figure 7:**
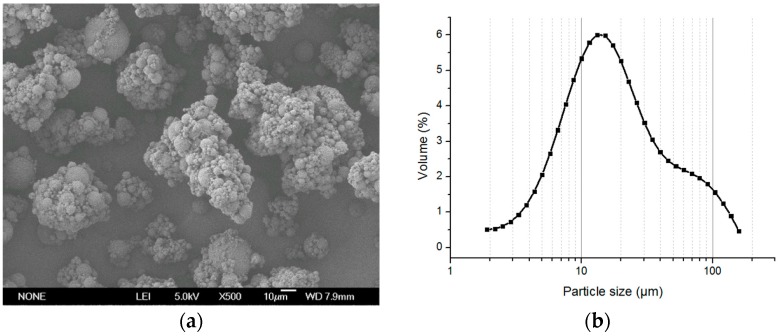
The photographs of the polymer microspheres of the (**a**) membrane emulsification (SEM ×500) and (**b**) size distribution (dispersed).

**Figure 8:**
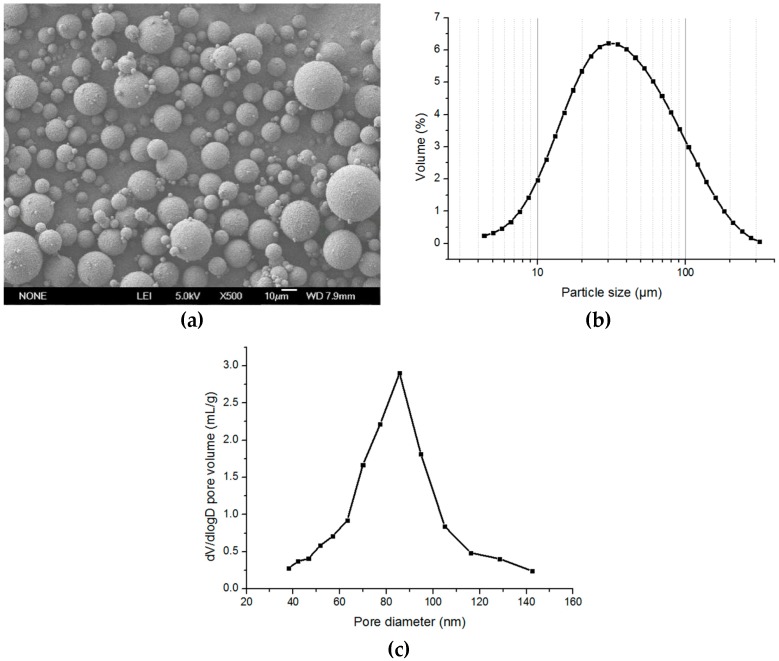
The photographs of the polymer microspheres by screen emulsification (**a**) SEM (×500); (**b**) size distribution; and (**c**) mercury porosimetry measurements.

In order to control the particle size, the membrane emulsification method was used. When using the 50-μm membrane, a large number of small particles formed, almost all of the small microspheres (about 15 μm) aggregated to form larger aggregations (Figure 7), and the pore size of the microspheres was about 100 nm. Considering that the largest pore size of the commercial membrane is 50 μm, a uniform screen with a hole size of around 300 μm was used in the first step of emulsification to increase the size of the oil droplets. After polymerization, the microspheres had a particle size of about 20 μm, and the aggregation was effectively inhibited (Figure 8).

**Figure 9:**
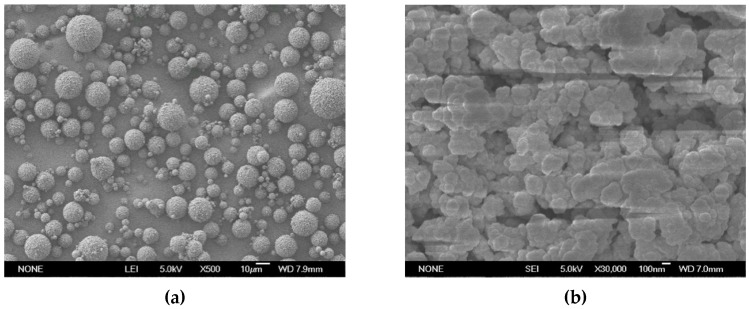

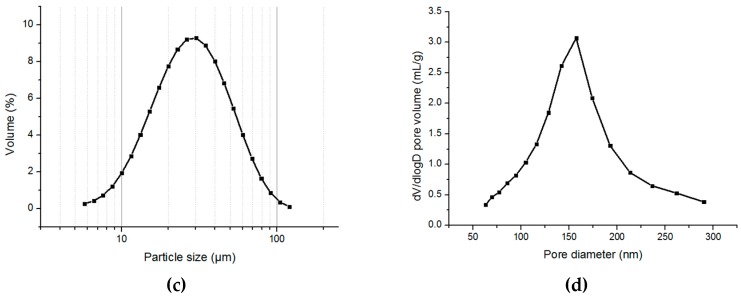
The photographs and mercury porosimetry measurements of the microspheres in optimized concentration of Span 80. (**a**) SEM (×500); (**b**) SEM (×30,000); (**c**) size distribution; and (**d**) mercury porosimetry measurements.

The pore size of the microspheres was about 90 nm (Figure 8c). Because the pore size was positively correlated to the amount of surfactant, we further optimized the content of Span 80 in the oil phase to control the porous structure. The pore characters with a different Span 80 is shown in Table 4. According to the table, when the content of Span 80 was 55% (based on the total amount of ST and DVB), the pore diameter was more than 150 nm (Figure 9, Table 4), which could satisfy the requirement of the separation and purification of large molecules. From the results, it can be concluded that the aggregation was reduced by utilizing the ATPS when excluding the effect of the small particles.

## 4. Conclusions

The weak aggregation of gigaporous polymer microspheres occurred when using the reverse micelle swelling method because of the disappearance of the interface between the water channel inside the oil droplet and the outside aqueous phase. A new method, called the aqueous two-phase system (ATPS), was herein proposed to solve the aggregation. A relatively stable boundary between the aqueous channel and the external water environment was established, which provided an interface onto which the stabilizer could adsorb.

The weak aggregation of gigaporous polymer microspheres were effectively reduced by the utilization of the ATPS. Based on the surfactant reverse micelles swelling method and the ATPS, we finally prepared the poly(styrene-divinyl benzene) particles with controllable particle sizes from 10–40 μm and pore sizes from 90–150 nm under the conditions of the screen emulsification method to make first emulsion, 120-rpm stirring for polymerization, and 55% surfactant to produce the pore. This method could reduce the post-treatment of the aggregations, especially for a large amount of products.

## Figures and Tables

**Table 1:** The recipe for microspheres preparation.

Materials of continuous phase	Weight (g)		Materials of dispersed phase	Weight (g)
	First step	Second step		
**PVA**	0.2–1.6	0.5–4.0	**ST**	3.0
**Water**	17.0–19.5	68.0–78.0	**DVB**	1.0
**PEG(*M*_w_ = 2,000, 6,000 and 20,000)**	0–0.6	0–3.0	**HD**	0.4
**Dextran**	0–0.6	0–3.0	**Span 80**	2.0–2.4
**Ammonium sulfate**	0–2.0	0–8.0	**BPO**	0.8
**NaCl**	0–2.0	0–8.0		

**Table 2:** The concentration of materials in the aqueous two-phase system (ATPS).

Concentration	PEG(*w*/*w*)	PVA(*w*/*w*)	PEG/PVA
*C*_inner_	1.6%	7.1%	0.23
*C*_outer_	3.5%	2.3%	1.52
*C*_total_	3.3%	2.8%	1.18

**Table 3:** The relationship between stirring rate/ swelling time and particle size.

Stirring rate (rpm)	Swelling time (min)	Average particle size (μm)	Uniformity of particle size	Average pore size (nm)	Yield (%)
150	10	10	0.52	80	87
70	10	25	0.33	130	91
70	5	350	0.45	110	93

**Table 4:** The pore characters with different Span 80.

Amount of Span 80 (%) (based on the total amount of ST and DVB)	Average pore size (nm)	Standard error of pore size (nm)	Total pore volume (mL/g)	Porosity (%)	Morphology (watch by SEM)
50	90	22.1	2.06	70	Porous
55	155	40.4	2.87	77	Gigaporous
60	165	48.1	3.60	81	Loose
